# Ten Simple Rules for Writing Research Papers

**DOI:** 10.1371/journal.pcbi.1003453

**Published:** 2014-01-30

**Authors:** Weixiong Zhang

**Affiliations:** Department of Computer Science and Engineering, Department of Genetics, Washington University in St. Louis, St. Louis, Missouri, United States of America; University of California San Diego, United States of America

The importance of writing well can never be overstated for a successful professional career, and the ability to write solid papers is an essential trait of a productive researcher. Writing and publishing a paper has its own life cycle; properly following a course of action and avoiding missteps can be vital to the overall success not only of a paper but of the underlying research as well. Here, we offer ten simple rules for writing and publishing research papers.

As a caveat, this essay is not about the mechanics of composing a paper, much of which has been covered elsewhere, e.g., [1], [2]. Rather, it is about the principles and attitude that can help guide the process of writing in particular and research in general. In this regard, some of the discussion will complement, extend, and refine some advice given in early articles of this Ten Simple Rules series of *PLOS Computational Biology* [3]–[8].

## Rule 1: Make It a Driving Force

Never separate writing a paper from the underlying research. After all, writing and research are integral parts of the overall enterprise. Therefore, design a project with an ultimate paper firmly in mind. Include an outline of the paper in the initial project design documents to help form the research objectives, determine the logical flow of the experiments, and organize the materials and data to be used. Furthermore, use writing as a tool to reassess the overall project, reevaluate the logic of the experiments, and examine the validity of the results during the research. As a result, the overall research may need to be adjusted, the project design may be revised, new methods may be devised, and new data may be collected. The process of research and writing may be repeated if necessary.

## Rule 2: Less Is More

It is often the case that more than one hypothesis or objective may be tackled in one project. It is also not uncommon that the data and results gathered for one objective can serve additional purposes. A decision on having one or more papers needs to be made, and the decision will be affected by various factors. Regardless of the validity of these factors, the overriding consideration must be the potential impact that the paper may have on the research subject and field. Therefore, the significance, completeness, and coherence of the results presented as a whole should be the principal guide for selecting the story to tell, the hypothesis to focus upon, and materials to include in the paper, as well as the yardstick for measuring the quality of the paper. By this metric, *less is more*, i.e., fewer but more significant papers serve both the research community and one's career better than more papers of less significance.

## Rule 3: Pick the Right Audience

Deciding on an angle of the story to focus upon is the next hurdle to jump at the initial stage of the writing. The results from a computational study of a biological problem can often be presented to biologists, computational scientists, or both; deciding what story to tell and from what angle to pitch the main idea is important. This issue translates to choosing a target audience, as well as an appropriate journal, to cast the main messages to. This is critical for determining the organization of the paper and the level of detail of the story, so as to write the paper with the audience in mind. Indeed, writing a paper for biologists in general is different from writing for specialists in computational biology.

## Rule 4: Be Logical

The foundation of “lively” writing for smooth reading is a sound and clear logic underlying the story of the paper. Although experiments may be carried out independently, the result from one experiment may form premises and/or provide supporting data for the next experiment. The experiments and results, therefore, must be presented in a logical order. In order to make the writing an easy process to follow, this logical flow should be determined before any other writing strategy or tactic is exercised. This logical order can also help you avoid discussing the same issue or presenting the same argument in multiple places in the paper, which may dilute the readers' attention.

An effective tactic to help develop a sound logical flow is to imaginatively create a set of figures and tables, which will ultimately be developed from experimental results, and order them in a logical way based on the information flow through the experiments. In other words, the figures and tables alone can tell the story without consulting additional material. If all or some of these figures and tables are included in the final manuscript, make every effort to make them self-contained (see Rule 5 below), a favorable feature for the paper to have. In addition, these figures and tables, as well as the threading logical flow, may be used to direct or organize research activities, reinforcing Rule 1.

## Rule 5: Be Thorough and Make It Complete

Completeness is a cornerstone for a research paper, following Rule 2. This cornerstone needs to be set in both content and presentation. First, important and relevant aspects of a hypothesis pursued in the research should be discussed with detailed supporting data. If the page limit is an issue, focus on one or two main aspects with sufficient details in the main text and leave the rest to online supporting materials. As a reminder, be sure to keep the details of all experiments (e.g., parameters of the experiments and versions of software) for revision, post-publication correspondence, or importantly, reproducibility of the results. Second, don't simply state what results are presented in figures and tables, which makes the writing repetitive because they are self-contained (see below), but rather, interpret them with insights to the underlying story to be told (typically in the results section) and discuss their implication (typically in the discussion section).

Third, make the whole paper self-contained. Introduce an adequate amount of background and introductory material for the right audience (following Rule 3). A statistical test, e.g., hypergeometric tests for enrichment of a subset of objects, may be obvious to statisticians or computational biologists but may be foreign to others, so providing a sufficient amount of background is the key for delivery of the material. When an uncommon term is used, give a definition besides a reference to it. Fourth, try to avoid “making your readers do the arithmetic” [9], i.e., be clear enough so that the readers don't have to make any inference from the presented data. If such results need to be discussed, make them explicit even though they may be readily derived from other data. Fifth, figures and tables are essential components of a paper, each of which must be included for a good reason; make each of them self-contained with all required information clearly specified in the legend to guide interpretation of the data presented.

## Rule 6: Be Concise

This is a caveat to Rule 5 and is singled out to emphasize its importance. Being thorough is not a license to writing that is unnecessarily descriptive, repetitive, or lengthy. Rather, on the contrary, “simplicity is the ultimate sophistication” [10]. Overly elaborate writing is distracting and boring and places a burden on the readers. In contrast, the delivery of a message is more rigorous if the writing is precise and concise. One excellent example is Watson and Crick's Nobel-Prize-winning paper on the DNA double helix structure [11] —it is only two pages long!

## Rule 7: Be Artistic

A complete draft of a paper requires a lot of work, so it pays to go the extra mile to polish it to facilitate enjoyable reading. A paper presented as a piece of art will give referees a positive initial impression of your passion toward the research and the quality of the work, which will work in your favor in the reviewing process. Therefore, concentrate on spelling, grammar, usage, and a “lively” writing style that avoids successions of simple, boring, declarative sentences. Have an authoritative dictionary with a thesaurus and a style manual, e.g., [1], handy and use them relentlessly. Also pay attention to small details in presentation, such as paragraph indentation, page margins, and fonts. If you are not a native speaker of the language the paper is written in, make sure to have a native speaker go over the final draft to ensure correctness and accuracy of the language used.

## Rule 8: Be Your Own Judge

A complete manuscript typically requires many rounds of revision. Taking a correct attitude during revision is critical to the resolution of most problems in the writing. Be objective and honest about your work and do not exaggerate or belittle the significance of the results and the elegance of the methods developed. After working long and hard, you are an expert on the problem you studied, and *you are the best referee of your own work, after all*. Therefore, inspect the research and the paper in the context of the state of the art.

When revising a draft, purge yourself out of the picture and leave your passion for your work aside. To be concrete, put yourself completely in the shoes of a referee and scrutinize all the pieces—the significance of the work, the logic of the story, the correctness of the results and conclusions, the organization of the paper, and the presentation of the materials. In practice, you may put a draft aside for a day or two—try to forget about it completely—and then come back to it fresh, consider it as if it were someone else's writing, and read it through while trying to poke holes in the story and writing. In this process, extract the meaning literally from the language as written and do not try to use your own view to interpret or extrapolate from what was written. Don't be afraid to throw away pieces of your writing and start over from scratch if they do not pass this “not-yourself” test. This can be painful, but the final manuscript will be more logically sound and better organized.

## Rule 9: Test the Water in Your Own Backyard

It is wise to anticipate the possible questions and critiques the referees may raise and preemptively address their concerns before submission. To do so, collect feedback and critiques from others, e.g., colleagues and collaborators. Discuss your work with them and get their opinions, suggestions, and comments. A talk at a lab meeting or a departmental seminar will also help rectify potential issues that need to be addressed. If you are a graduate student, running the paper and results through the thesis committee may be effective to iron out possible problems.

## Rule 10: Build a Virtual Team of Collaborators

When a submission is rejected or poorly reviewed, don't be offended and don't take it personally. Be aware that the referees spent their time on the paper, which they might have otherwise devoted to their own research, so they are doing you a favor and helping you shape the paper to be more accessible to the targeted audience. Therefore, consider the referees as your collaborators and treat the reviews with respect. This attitude can improve the quality of your paper and research.

Read and examine the reviews objectively—the principles set in Rule 8 apply here as well. Often a criticism was raised because one of the aspects of a hypothesis was not adequately studied, or an important result from previous research was not mentioned or not consistent with yours. If a critique is about the robustness of a method used or the validity of a result, often the research needs to be redone or more data need to be collected. If you believe the referee has misunderstood a particular point, check the writing. It is often the case that improper wording or presentation misled the referee. If that's the case, revise the writing thoroughly. Don't argue without supporting data. Don't submit the paper elsewhere without additional work. This can only temporally mitigate the issue, you will not be happy with the paper in the long run, and this may hurt your reputation.

Finally, keep in mind that writing is personal, and it takes a lot of practice to find one's style. What works and what does not work vary from person to person. Undoubtedly, dedicated practice will help produce stronger papers with long-lasting impact.
